# No Association between *TGFB1* Polymorphisms and Late Radiotherapy Toxicity: A Meta-Analysis

**DOI:** 10.1371/journal.pone.0076964

**Published:** 2013-10-09

**Authors:** Mei-Ling Zhu, MengYun Wang, Ting-Yan Shi, Qiao-Xin Li, Pan Xi, Kai-Qin Xia, Leizhen Zheng, Qing-Yi Wei

**Affiliations:** 1 Department of Oncology, Xin Hua Hospital affiliated To Shanghai Jiaotong University School of Medicine, Shanghai, China; 2 Cancer Institute, Fudan University Shanghai Cancer Center, Fudan University, Shanghai, China; 3 Department of Pathology, Fudan University Shanghai Cancer Center, Fudan University, Shanghai, China; 4 Department of Epidemiology, the University of Texas MD Anderson Cancer Center, Houston, Texas, United States of America; Northwestern University Feinberg School of Medicine, United States of America

## Abstract

**Background:**

Transforming growth factor-beta 1 (TGF-β1) protein may be multifunctional and related to the development of fibrosis, induction of apoptosis, extracellular signaling and inhibition of proliferation in response to radiation-induced DNA damage. Several studies have investigated associations between single nucleotide polymorphisms (SNPs) in the *TGFB1* gene and risk of late radiation-induced injury of normal tissue, but the conclusions remain controversial.

**Methods:**

We searched three electronic databases (i.e., MEDLINE, EMBASE and EBSCO) for eligible publications and performed a meta-analysis assessing the association of three commonly studied SNPs in *TGFB1* (i.e., rs1800469, rs1800470 and rs1800471) with risk of late radiation-induced injury of normal tissue.

**Results:**

We finally included 28 case-only studies from 16 publications on aforementioned SNPs in *TGFB1*. However, we did not find statistical evidence of any significant association with overall risk of late radiotherapy toxicity in the pooled analysis or in further stratified analysis by cancer type, endpoint, ethnicity and sample size.

**Conclusions:**

This meta-analysis did not find statistical evidence for an association between SNPs in *TGFB1* and risk of late radiation-induced injury of normal tissue, but this finding needs further confirmation by a single large study.

## Introduction

Radiotherapy remains a cornerstone of modern cancer management, with curative or palliative potential in roughly half of incident solid tumors, and has the advantage of organ and function preservation in most cases [1]. Unfortunately, radiation-induced injury of normal tissue limits deliverable intensity of radiotherapy and influences the long-term health-related quality of life for patients [1]. During the last decade, researchers have made great efforts to find reliable and clinically useful assays for predicting individual risk of normal tissue complication prior to the radiotherapy, which has been referred to as ‘the Holy Grail of radiobiology’ [2]. There is a growing consensus that the risk of normal tissue complication is influenced by single nucleotide polymorphisms (SNPs), the most common genetic variations, which could contribute to patient susceptibility to adverse radiotherapy effects [3]. 

A number of studies have reported associations between various SNPs and radiation-induced injury of normal tissue [3,4], among which the most extensively studied were SNPs in *TGFB1*. The human *TGFB1* gene is located at chromosome 19q13.1 and encodes the transforming growth factor-beta1 (TGF-β1) protein, a versatile pro-fibrotic cytokine that is involved in the development and continuation of post-irradiation injury in various tissues [5]. The TGF-β1 protein has effects on the pathological accumulation of the extracellular matrix that accompanies inflammatory and fibrotic diseases such as glomerulonephritis [1]. TGF-β1 also stimulates the differentiation of fibroblasts and inhibits epithelial repair [1]. Circulating TGF-β1 levels are known to be up-regulated in various irradiated normal tissues at the early phase of fibrogenesis, which is regarded as a wound healing response [6]. Associations between increased circulating levels of TGF-β1 and radiation-induced fibrosis have been reported in patients with various types of cancer, such as cancers of the lung, breast and head-and-neck [6]. The TGF-β1 protein may also contribute to the development of fibrosis, extracellular signaling, inducing apoptosis and inhibiting proliferation in response to radiation-induced DNA damage [1]. Some SNPs in *TGFB1* have been reported to affect TGF-β1 levels in circulating plasma and hence lead to the risk of developing radiation-induced toxicity [7]. For example, the -509T>C SNP in the promoter was found to be significantly associated with an increased circulating concentration of TGF-β1 [8], whereas the Arg25Pro SNP in the exon 1 was reported to be associated with inter-individual variation in levels of the TGF-β1 production *in vitro* and also with fibrosis in lung allografts [5]. For the Pro10Leu SNP, the 10Pro allele was found to be associated with increased TGF-β1 secretion rates [9,10]. Thus, SNPs in *TGFB1* represent logical candidates for association studies designed to address late toxicity following radiotherapy. 

To date, at least 638 SNPs in the *TGFB1* gene have been reported according to the dbSNP database (http://www.ncbi.nlm.nih.gov/projects/SNP), among which three common SNPs, rs1800469 c.509TC in the 5’near gene, rs1800470 c.29CT encoding Pro10Le and rs1800471 c.74GC encoding Arg25Pro, have been widely studied for associations with risk of late radiotherapy toxicity, but the results are mixed rather than conclusive, partially because of possible weak effects of these SNPs on late radiotherapy toxicity risk or relatively small studies to detect such weak associations. Therefore, we performed a comprehensive meta-analysis that improved statistical power to derive a more precise risk estimate for the associations.

## Material and Methods

### Identification and eligibility of relevant studies

We searched three electronic databases (i.e., MEDLINE, EMBASE and EBSCO) for all relevant publications using the following terms: “*TGFB1*” or “TGFbeta1” or “transforming growth factor beta 1”, “genetic variation” or “polymorphism”, “cancer” or “neoplasm” or “tumor” or “malignancy”, “radiotherapy” or “radiation”, “toxicity” or “adverse effect” (as of April 15, 2013). We also manually examined the references of the retrieved articles and relevant reviews for additional relevant eligible studies. We contacted authors directly for crucial rude data that were unavailable in the original publications. 

We defined the inclusion and exclusion criteria as follows: evaluation of *TGFB1* SNPs (i.e., rs1800469, rs1800470, and rs1800471) and late radiotherapy toxicity; written in English or Chinese; sufficient information provided to estimate odds ratios (ORs) and their 95% confidence intervals (CIs). We did not consider abstracts or unpublished reports. We also excluded investigations of *TGFB1* SNPs with acute radiotherapy toxicity. Late radiotherapy toxicities occur in months to years after treatment, including fibrosis, atrophy, vascular damage, neural damage as well as a range of endocrine and growth-related effects. Acute effects typically emerge within a few weeks of the completion of a course of fractionated radiotherapy, including skin erythema, dry or moist desquamation of the skin, mucositis, nausea and diarrhea., Additionally, if studies had the same subjects or overlapping data, we only included the latest or the most complete study with the largest sample size in this meta-analysis.

### Data extraction

Two authors independently extracted data from each study and reached a consensus on all items, including first author's surname, year of publication, country of origin, ethnicity, cancer type, total number of genotyped cases, genotyping methods, endpoints, scoring system, treatment summary, and numbers of genotypes for each SNP in cases. For studies that included subjects of a different racial descent, we extracted data separately for each ethnic group and categorized these groups as Caucasians, Asian and Mixed (which contained more than one ethnic group). In addition, studies involving different endpoints were divided into multiple single studies for subgroup analyses.

### Statistical methods

We evaluated associations between *TGFB1* SNPs and risk of late radiotherapy toxicity by the pooled ORs with the corresponding 95% CIs for dominant models of rs1800469 (TC/CC vs. TT), rs1800470 (TC/TT vs. CC) and rs1800471 (GC/CC vs. GG). We also performed stratification analyses by cancer type (breast cancer, prostate cancer and others, but if one cancer type had been investigated in less than two studies, we merged it into the ‘‘others’’ category), ethnicity (Caucasian and others), endpoint (fibrosis, breast appearance, gastrointestinal morbidity, genitourinary morbidity and others) and sample size (numbers of cases < 400, ≥ 400). We performed Chi square-based Q-test to assess between-study heterogeneity and considered it significant if *P* < 0.10. When the *P* value of the heterogeneity test was ≥ 0.10, we used the fixed-effects model (Mantel-Haenszel method), assuming no significant heterogeneity of effect size across all studies [11]. Otherwise, we chose the random-effects model (DerSimonian and Laird method), which tends to provide wider 95% CIs because the constituent studies differ among themselves [12]. In addition, we estimated the potential publication bias by the inverted funnel plot and Egger’s linear regression test; an asymmetric plot or *P* < 0.05 determined by Egger’s test suggested a possible publication bias [13]. If publication bias existed, we adjusted for it by using the Duval and Tweedie nonparametric ‘‘trim and fill’’ method [14]. Finally, we performed sensitivity analyses to assess the effects of individual studies on overall cancer risk by excluding each study individually each time and recalculating the ORs and 95% CIs. We conducted all analyses with STATA software (version 11.0; Stata Corporation, College Station, TX). All *P* values were two-sided with a significance level of *P* < 0.05. 

## Results

### Study characteristics

**Figure 1:**
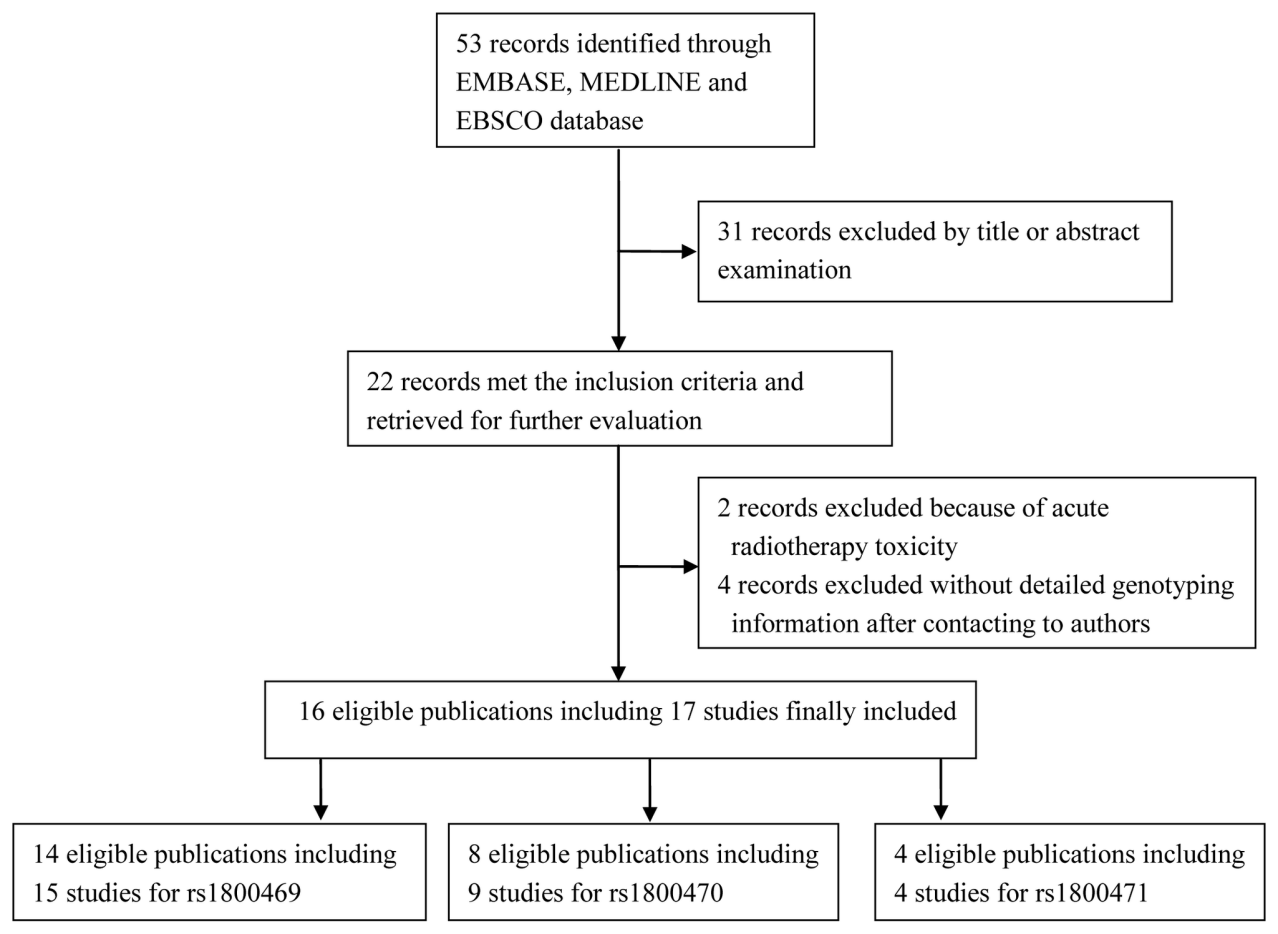
Flow chart of included studies for this meta-analysis.

We identified a total of 53 relevant publications after initial screening, of which 22 met the inclusion criteria and were subjected for further evaluation. We excluded four publications with conference abstracts or insufficient data to extract ORs and 95% CIs (after failure to get a response from the authors) [15-18]. We also excluded two publications that investigated the associations of *TGFB1* SNPs with acute radiotherapy toxicity [19,20]. Finally, this meta-analysis included 16 publications [15,21-35] with 17 case-only studies, because we considered one publication [21] with two endpoints as two independent studies. We identified 15 studies for rs1800469, nine studies for rs1800470, and four studies for rs1800471 (Figure **1**). For rs1800469, nine studies focused on breast cancer, four on prostate cancer, and two on other cancers. There were four studies on the endpoint of fibrosis, three studies on breast appearance, two studies on gastrointestinal morbidity, two studies on genitourinary morbidity, and four studies on four other endpoints. Among all studies, 12 studies were for Caucasians, one for Asians and three for mixed ethnic groups. Additionally, 12 studies used a sample size < 400 and three studies ≥ 400. For 1800470, three studies focused on breast cancer, three on prostate cancer, and three on other cancers. There were three studies on fibrosis, two studies on gastrointestinal morbidity, and four studies on other endpoints. Overall, eight studies used Caucasians, and one used mixed ethic groups. Seven studies used a sample size < 400 and two studies ≥ 400. In addition, there were four studies that investigated rs1800471 SNP (Table **1**). 

**Table 1:** Characteristics of studies included in the meta-analysis.

Author, year	Country	Ethnicity	Cancer type	Sample size	Genotype method	Endpoint	Scoring system	Treatment summary	Polymorphism
Quarmby, 2003	UK	Caucasian	Breast cancer	103	PCR–RFLP	Fibrosis	LENT/SOMA	RT	rs1800469, rs1800470, rs1800471
Andreassen, 2003	Denmark	Caucasian	Breast cancer	41	SNaPshot™	Fibrosis, Telangiectasia	LENT/SOMA	S,RT,H	rs1800469
Andreassen, 2005	UK	Caucasian	Breast cancer	52	TaqMan	Breast appearance	Three-point scale	S,CRT,H	rs1800469
De Ruyck, 2006	Belgium	Caucasian	Gynecologic cancers	78	PCR-RFLP	Various effects	CTCAEv3.0	S,CRT,H	rs1800469, rs1800470, rs1800471
Andreassen, 2006	Denmark	Caucasian	Breast cancer	120	TaqMan	Fibrosis	LENT/SOMA	S,CRT	rs1800469
Damaraju, 2006	Canada	Mixed	Prostate cancer	83	Pyrosequencing	Late bladder or rectal toxicity	RTOG	S,RT,H	rs1800471
Giotopoulos, 2007	UK	Mixed	Breast cancer	167	PCR-RFLP	Fibrosis, telangiectasia	SOMA/RTOG	S,CRT,H	rs1800469
Peters, 2008	USA	Mixed	Prostate cancer	141	Sequencing	Rectal bleeding	RTOG	RT,H	rs1800469, rs1800470, rs1800471
Suga, 2008	Japan	Asian	Prostate cancer	197	MassARRAY	Dysuria	LENT/SOMA	RT,H	rs1800469
Azria, 2008	Swiss	Caucasian	Miscellaneous cancers	34	Surveyor nuclease assay	Various effects	RTOG/EORTC	RT	rs1800469, rs1800470
Alsbeih, 2010	Saudi Arabia	Caucasian	Nasopharyngeal cancer	60	Sequencing	Fibrosis	RTOG/EORTC	CRT	rs1800470
Barnett, 2010	UK	Caucasian	Breast cancer	778	Taqman	Breast appearance	Three-point scale	S,CRT,H	rs1800469
Martin, 2010	UK	Caucasian	Breast cancer	190	Sequencing	Breast appearance	Three-point scale	S,CRT,H	rs1800469, rs1800470, rs1800471
Zschenker, 2010	Germany	Caucasian	Breast cancer	69	PCR–RFLP	Fibrosis	LENT/SOMA	S,CRT,H	rs1800469
Terrazzino, 2012	Italy	Caucasian	Breast cancer	257	PCR–RFLP	Fibrosis	LENT/SOMA	S,CRT,H	rs1800469, rs1800470
Fachal, 2012	Spain	Caucasian	Prostate cancer	413	SNaPshot™	Gastrointestinal morbidity	CTCAEv3.0	CRT,H	rs1800469, rs1800470

PCR-RFLP, polymerase chain reaction-restriction fragment length polymorphism; RT, radiotherapy; CRT, chemoradiotherapy; S, surgery; H, hormonotherapy; RTOG/ EORTC, radiation therapy oncology Group/European Organization for Research and Treatment of Cancer; LENT/SOMA, late effects of Normal Tissue/Subjective objective management analytical; CTCAEv3.0, common terminology criteria for adverse events version 3.0; some of the studies were marked as ‘mixed’ ethnic, because the genotyping data was mixed from different populations.

### Meta-analysis results

**Figure 2:**
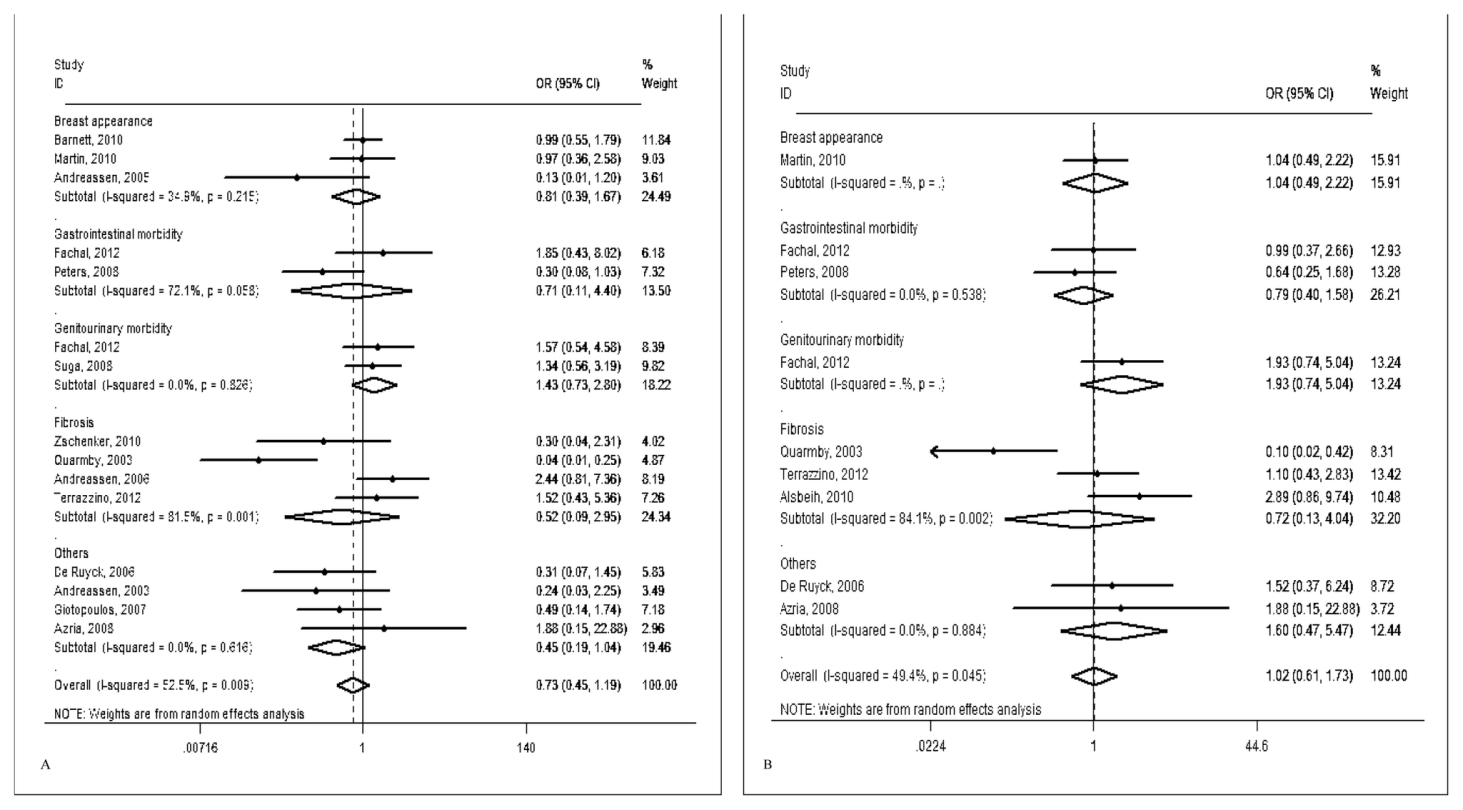
Forest plot for late radiotherapy toxicity risk of different endpoints associated with *TGFB1* SNPs. (A) rs1800469; (B) rs1800470. For each study, the estimates of OR and its 95% CI are plotted with a box and a horizontal line. The symbol filled diamond indicates pooled OR and its 95% CI.

For the rs1800469 SNP, we obtained genotyping data for 2,926 cases from 15 studies. We found no association between the rs1800469 SNP and late radiotherapy toxicity risk, either in the overall pooled meta-analysis (TC/CC vs. TT: OR = 0.73, 95% CI = 0.45-1.19, *P* = 0.206) or in the stratified analysis by ethnicity, cancer type, endpoint, and sample size. For rs1800470, we included a total of 1,669 cases from nine publications, and we also did not find any statistical evidence of significant associations of the SNP with late radiotherapy toxicity risk in both the pooled analyses (TC/TT vs. CC: OR = 1.02, 95% CI = 0.61-1.73, *P* = 0.932) and further subgroup analyses (Table **2**, Figure **2**). For rs1800471, we included 455 cases from four studies and found no significant association of the SNP with late toxicity risk (GC/CC vs. GG: OR = 1.37, 95% CI = 0.76-2.45, *P* = 0.297). Because we included only a limited number of publications for this SNP, we did not conduct further stratified analysis.

**Table 2:** Meta-analysis of the association between *TGFB1* SNPs and late radiotherapy toxicity.

Variables	No. of studies	rs1800469 TC/CC vs. TT			No. of studies	rs1800470 TC/TT vs. CC		
		OR (95% CI)^a^	*P*_OR_^b^	*P*_het_^c^		OR (95% CI)^a^	*P*_OR_^b^	*P*_het_^c^
All	15	0.73 (0.45-1.19)	0.206	0.009	9	1.02 (0.61-1.73)	0.932	0.045
Cancer type								
Breast cancer	9	0.59 (0.29-1.20)	0.145	0.007	3	0.56 (0.16-1.91)	0.351	0.013
Prostate cancer	4	1.06 (0.49-2.26)	0.887	0.146	3	1.07 (0.57-2.02)	0.830	0.277
Others	2	0.58 (0.11-3.16)	0.529	0.225	3	2.16 (0.91-5.12)	0.081	0.788
Endpoint								
Fibrosis	4	0.52 (0.09-2.95)	0.463	0.001	3	0.72 (0.13-4.05)	0.705	0.002
Breast appearance	3	0.81 (0.40-1.67)	0.574	0.215	1	1.04 (0.49-2.22)	0.925	/
Gastrointestinal morbidity	2	0.71 (0.12-4.40)	0.713	0.058	2	0.79 (0.40-1.58)	0.511	0.538
Genitourinary morbidity	2	1.43 (0.73-2.80)	0.302	0.826	1	1.93 (0.74-5.04)	0.181	/
Others	4	0.45 (0.19-1.04)	0.063	0.616	2	1.60 (0.47-5.47)	0.457	0.884
Ethnicity								
Caucasian	12	0.75 (0.42-1.35)	0.342	0.010	8	1.10 (0.61-1.97)	0.761	0.041
Others	3	0.63 (0.25-1.64)	0.347	0.115	1	0.64 (0.25-1.68)	0.368	/
Sample Size								
< 400	12	1.17 (0.72-1.90)	0.529	0.618	7	0.92 (0.46-1.81)	0.797	0.030
≥ 400	3	0.57 (0.30-1.07)	0.080	0.006	2	1.40 (0.70-2.78)	0.344	0.343
Publication Bias ^d^			0.072				0.815	

^a^ Random effects model.

^b^ *P* value of the Z-test for odds ration test.

^c^ *P* value of the Q-test for heterogeneity test.

^d^ *P* value of Egger's test for publication bias.

### Heterogeneity and sensitivity analyses

Because substantial heterogeneities were observed among studies for the associations between the two SNPs (i.e., rs1800469 and rs1800470) and late radiotherapy toxicity (*P* = 0.009 and *P* = 0.045 for the heterogeneity tests, respectively), we used the random-effects model that generated wider CIs. For rs1800471, we did not find evidence of heterogeneity among studies (*P* = 0.912) and then used the fixed-effects model. The leave-one-out sensitivity analysis indicated that no single study changed the pooled ORs qualitatively (data not shown).

### Publication bias

The shapes of the funnel plots seemed symmetrical, indicating that there was no obvious publication bias for associations between the *TGFB1* SNPs (i.e., rs1800469, rs1800470 and rs1800471) and late radiotherapy toxicity risk. Egger’s test provided further statistical evidence indicating that no publication bias existed in this meta-analysis (Egger’s test for rs1800469: *P* = 0.072; for rs1800470: *P* = 0.815 and for rs1800471: *P* = 0.744). 

## Discussion

Our meta-analysis comprehensively explored the associations between *TGFB1* SNPs and the risk of late radiotherapy toxicity. We found that none of the three SNPs (i.e., rs1800469, rs1800470 and rs1800471) was significantly associated with late toxicity risk in the pooled analysis. Similarly, in the subgroup analysis, we found no statistical association between the SNPs and late toxicity risk in any of the subgroups. 

TGF-β1 is a multifunctional cytokine associated with proliferation, differentiation, and deposition of extracellular matrix proteins in irradiated cells [36]. Considerable evidence shows that TGF-β1 acts as a key mediator of fibrosis by recruiting inflammatory cells and activating fibroblasts to produce the extracellular matrix. TGF-β1 is central in the mitigation of post-irradiation injury in various normal tissues and tumor cells [37]. It has been observed that a dose as low as 0.1 Gy of ionizing radiation could directly activate TGF-β1 in less than 1 hour [38,39]. Additionally, ionizing radiation indirectly induces TGF-β1 activation by damaging endothelial cells and alters the homeostasis of reactive oxygen and nitrogen species [1]. Elevated plasma TGF-β1 levels have been studied in lung cancer patients to predict patients’ risk of developing radiation-induced pulmonary fibrosis. Some studies reported a correlation between elevated serum TGF-β1 levels and increased risk of fibrosis after cancer radiotherapy [40]. Patients heterozygous for the SNP rs1800469 (C/T) had significantly higher TGF-β1 production, and other SNPs were also found to be correlated with elevated TGF-β1 levels [41]. Due to a relatively strong linkage disequilibrium, the SNP rs1800469 T allele and rs1800470 codon 10 C (minority) allele are often inherited together. 

To date, two reported meta-analyses have focused on associations between *TGFB1* SNPs and risk of late radiotherapy toxicity [3,7]. Andreassen et al. performed one meta-analysis of 13 published studies with 1880 participants addressing the impact of the rs1800469 SNP on risk of late toxicity in general with an emphasis on the breast [3]. In both cases, the analyses showed a significantly increased risk of late toxicity in patients carrying the TT genotype, corresponding to a small increment in risk of late toxicity associated with the T allele. Nevertheless, it is not uncommon to observe that small studies were reported with high ORs, larger studies with borderline findings, and some with even an inverse association, suggesting some possible publication bias. Therefore, even though *TGFB1* SNPs are among the most extensively studied SNPs in terms of sensitivity of normal tissues to radiobiology, it is difficult to draw any definite conclusions [3]. Later on, Barnett et al. obtained data on the *TGFB1* SNP rs1800469 genotype, as well as treatment-related outcome data, and clinically-assessed fibrosis (measured at least two years after therapy) in 2782 participants from 11 patient cohorts, but their meta-analysis did not confirm previous reports of associations between fibrosis or overall toxicity and the rs1800469 SNP in breast cancer patients [7], which is consistent with our findings in our meta-analysis with further expanded sample size and improved statistical power. Our meta-analysis should generate a more precise risk estimate for the associations but still had a limited statistical power to detect a weak effect. For example, our present analysis had a 63.5 % power to detect OR= 1.42 as reported by Andreassen et al [3].

Notably, the results of the present meta-analysis require some caution in the interpretation, because of several limitations in the present and published meta-analyses. First, some data were excluded from the analyses, because of unavailable original data, which could cause some bias in the estimates. In addition, although there is no obvious evidence of publication bias observed in the meta-analysis, the power of the funnel plot to test the asymmetry with less than thirty publications is relatively low. Furthermore, most of the data on publication bias is retrospective rather than prospective, including our present analysis. Reporting publication bias from prospective studies is needed. Second, in the process of quality assessment and data extraction from the original literature, we found some studies that had overly small sample sizes, which may have resulted in false-positive or false-negative results for each study. Third, it has been reported that treatment-related factors, such as with or without chemotherapy or chemotherapy sequential may influence the outcome. However, due to the retrospective nature of the studies included in the meta-analysis, we could not obtain the needed information for assessing the effect of confounding factors on the outcome. Fourth, all included studies were of case-only design, which may have selection biases, implementation biases, and confounding bias, due to the nature of hospital-based studies. Fifth, radiotherapy toxicity is a complex phenotype involving many different pathological mechanisms, and these different processes may lead to various clinical end-points. Despite a recognized need for a standardized approach for reporting radiation toxicity, a variety of scoring systems have been used, and the toxicity remains generally under-reported. Sixth, according to the stratified analysis, we observed that the source of homogeneity may come from different cancer type, endpoint, ethnicity and sample size of each study. The results of subgroup analysis demonstrated that there was some variability among studies for the same endpoint. A possible explanation for this variability may be related to other unmeasured differences in the current analysis, such as the side effects scoring system or the time between radiotherapy and when the presence/absence of side effects occurred. Seventh, in most publications that were included in the meta-analysis, although the side effects and the corresponding risk factors were described, the information about the time between radiotherapy and when the presence/absence of side effects was often missing which could have contributed to the bias in estimating the true association and thus the influence on the outcome. Finally, some of the findings in subgroups may have been underestimated, because there was only one trail available.

## Conclusions

In conclusion, the present meta-analysis suggests that SNPs in *TGFB1* may not contribute to risk of late radiation-induced injury of normal tissue. Because the subjects from studies included in this meta-analysis are still nor large enough, well-designed prospective studies with larger sample sizes and more detailed information on confounding factors are required to validate these findings. 

## Supporting Information

Checklist S1 **PRISMA checklist.**
(DOC)
