# Supplementary material for: No Association between TGFB1 Polymorphisms and Late Radiotherapy Toxicity: A Meta-Analysis
Source: PLoS One. 2013 Oct 9;8(10):e76964. doi: 10.1371/journal.pone.0076964 (PMC3793936; doi:10.1371/journal.pone.0076964)
Supplement: Checklist S1 — PRISMA checklist. (DOC) [file pone.0076964.s001.doc]

| **Section/topic** | **#** | **Checklist item** | **Reported on page #** |
| --- | --- | --- | --- |
| **TITLE** | | |  |
| Title | 1 | No association between *TGFB1* polymorphisms and late radiotherapy toxicity: a meta-analysis | Title |
| **ABSTRACT** | | |  |
| Structured summary | 2 | Background; Methods; Results; Conclusions | Abstract |
| **INTRODUCTION** | | |  |
| Rationale | 3 | Three common SNPs in *TGFB1*, rs1800469 c.509T>C in the 5’near gene, rs1800470 c.29C>T encoding Pro10Le and rs1800471 c.74G>C encoding Arg25Pro, have been widely studied for their associations with risk of late radiotherapy toxicity, but the results are mixed rather than conclusive, partially because of possible weak effects of these SNPs on late radiotherapy toxicity risk or relatively small studies to detect such weak associations. | Introduction |
| Objectives | 4 | We performed a comprehensive meta-analysis that improves statistical power to derive a more precise risk estimate for the associations of three common SNPs in *TGFB1* with late radiotherapy toxicity risk. | Introduction |
| **METHODS** | | |  |
| Protocol and registration | 5 | No | / |
| Eligibility criteria | 6 | We defined inclusion and exclusion criteria as follows: evaluation of TGFB1 SNPs (i.e., rs1800469, rs1800470 and rs1800471) and late radiotherapy toxicity; written in English or Chinese; sufficient information provided to estimate odds ratios (ORs) and their 95% confidence intervals (CIs). We did not consider abstracts or unpublished reports. We also excluded investigations of TGFB1 SNPs with acute radiotherapy toxicity. | Identification and eligibility of relevant studies |
| Information sources | 7 | We searched three electronic databases (i.e., MEDLINE, EMBASE and EBSCO) for all relevant publications (as of April 15, 2013). | Identification and eligibility of relevant studies |
| Search | 8 | “TGFB1” or “TGFbeta1” or “transforming growth factor beta 1”, “genetic variation” or “polymorphism”, “cancer” or “neoplasm” or “tumor” or “malignancy”, “radiotherapy” or “radiation”, “toxicity” or “adverse effect” | Identification and eligibility of relevant studies |
| Study selection | 9 | We selected case-only studies that investigated the associations of TGFB1 SNPs with late radiotherapy toxicity. | Identification and eligibility of relevant studies |
| Data collection process | 10 | The credentials of the two investigators ZML and WMY are indicated in the author list. We searched three electronic databases (i.e., MEDLINE, EMBASE and EBSCO) for all relevant publications” (as of April 15, 2013). We also manually examined references of the retrieved articles and relevant reviews for additional relevant eligible studies. We contacted authors directly for crucial rude data, which were unavailable in original publications. | Data extraction |
| Data items | 11 | Two authors independently extracted data from each study and reached a consensus on all of the items, including first author's surname, year of publication, country of origin, ethnicity, cancer type, total number of genotyped cases, genotyping methods, endpoints, scoring system, treatment summary and numbers of genotypes for each SNP in cases. | Data extraction |
| Risk of bias in individual studies | 12 | We estimated the potential publication bias by the inverted funnel plot and Egger’s linear regression test; an asymmetric plot or P < 0.05 determined by Egger’s test suggested a possible publication bias | Statistical methods |
| Summary measures | 13 | We evaluated associations between TGFB1 SNPs and risk of late radiotherapy toxicity by the pooled ORs with the corresponding 95% CIs. | Statistical methods |
| Synthesis of results | 14 | We performed Chi square-based Q-test to assess the between-study heterogeneity and considered it significant if P < 0.10. When P value of the heterogeneity test was ≥ 0.10, we used the fixed-effects model (Mantel-Haenszel method); otherwise, we chose the random-effects model (DerSimonian and Laird method). | Statistical methods |

Page 1 of 2

| **Section/topic** | **#** | **Checklist item** | **Reported on page #** |
| --- | --- | --- | --- |
| Risk of bias across studies | 15 | We estimated the potential publication bias by the inverted funnel plot and the Egger’s linear regression test, and an asymmetric plot or P < 0.05 determined by Egger’s test suggests a possible publication bias. | Statistical methods |
| Additional analyses | 16 | We performed sensitivity analyses to assess the effect of individual studies on overall cancer risk by excluding each study individually and recalculating the ORs and 95% CIs. | Statistical methods |
| **RESULTS** | | |  |
| Study selection | 17 | We identified a total of 53 relevant publications after initial screening, of which 22 publications met the inclusion criteria and were subjected for further evaluation. | Study characteristics |
| Study characteristics | 18 | Each study characteristic was shown in Table 1. | Study characteristics |
| Risk of bias within studies | 19 | Present data on risk of bias of each study and, if available, any outcome level assessment (see item 12). | Study characteristics |
| Results of individual studies | 20 | Results of individual studies were shown in Figure 2, ideally with a forest plot. | Meta-analysis results |
| Synthesis of results | 21 | We found no association between the three SNPs in TGFB1 and late radiotherapy toxicity risk, when all eligible studies were pooled into the meta-analysis | Meta-analysis results |
| Risk of bias across studies | 22 | The shapes of the funnel plots seemed symmetrical, indicating that there was no obvious publication bias for associations between TGFB1 SNPs (i.e., rs1800469, rs1800470 and rs1800471) and late radiotherapy toxicity risk. Egger’s test provided further statistical evidence that no publication bias existed in this meta-analysis (the Egger’s test for rs1800469: P = 0.072; for rs1800470: P = 0.815 and for rs1800471: P = 0.744). | Publication bias |
| Additional analysis | 23 | Because substantial heterogeneities were observed among studies for the associations between the two SNPs (i.e., rs1800469 and rs1800470) and late radiotherapy toxicity (P = 0.009 and P = 0.045 for heterogeneity test, respectively), we used the random-effects model that generated wider CIs. For rs1800471, no heterogeneity was found among studies (P = 0.912) and the fixed-effects model was performed. The leave-one-out sensitivity analysis indicated that no single study changed the pooled ORs qualitatively | Heterogeneity and sensitivity analyses |
| **DISCUSSION** | | |  |
| Summary of evidence | 24 | We found no statistical associations between the SNPs in TGFB1 and late toxicity risk. These main findings would be helpful to healthcare providers. | Discussion |
| Limitations | 25 | First, some data were excluded from the analyses, because of unavailable original data, which could cause some bias in the estimates. In addition, although there is no obvious evidence of publication bias observed in the meta-analysis, the power of the funnel plot to test the asymmetry with less than thirty publications is relatively low. Furthermore, most of the data on publication bias is retrospective rather than prospective, including our present analysis. Reporting publication bias from prospective studies is needed. Second, in the process of quality assessment and data extraction from the original literature, we found some studies that had overly small sample sizes, which may have resulted in false-positive or false-negative results for each study. Third, it has been reported that treatment-related factors, such as with or without chemotherapy or chemotherapy sequential may influence the outcome. However, due to the retrospective nature of the studies included in the meta-analysis, we could not obtain the needed information for assessing the effect of confounding factors on the outcome. Fourth, all included studies were of case-only design, which may have selection biases, implementation biases, and confounding bias, due to the nature of hospital-based studies. Fifth, radiotherapy toxicity is a complex phenotype involving many different pathological mechanisms, and these different processes may lead to various clinical end-points. Despite a recognized need for a standardized approach for reporting radiation toxicity, a variety of scoring systems have been used, and the toxicity remains generally under-reported. Sixth, according to the stratified analysis, we observed that the source of homogeneity may come from different cancer type, endpoint, ethnicity and sample size of each study. The results of subgroup analysis demonstrated that there was some variability among studies for the same endpoint. A possible explanation for this variability may be related to other unmeasured differences in the current analysis, such as the side effects scoring system or the time between radiotherapy and when the presence/absence of side effects occurred. Seventh, in most publications that were included in the meta-analysis, although the side effects and the corresponding risk factors were described, the information about the time between radiotherapy and when the presence/absence of side effects was often missing which could have contributed to the bias in estimating the true association and thus the influence on the outcome. Finally, some of the findings in subgroups may have been underestimated, because there was only one trail available. | Discussion |
| Conclusions | 26 | In conclusion, the present meta-analysis suggests that SNPs in TGFB1 may not contribute to risk of late radiation-induced injury of normal tissue. Because the subjects from studies included in this meta-analysis are still nor large enough, well-designed prospective studies with larger sample sizes and more detailed information on confounding factors are required to validate these findings. | Conclusions |
| **FUNDING** | | |  |
| Funding | 27 | This study was supported by the funds from “China’s Thousand Talents Program” Recruitment at Fudan University. |  |

*From:*  Moher D, Liberati A, Tetzlaff J, Altman DG, The PRISMA Group (2009). Preferred Reporting Items for Systematic Reviews and Meta-Analyses: The PRISMA Statement. PLoS Med 6(6): e1000097. doi:10.1371/journal.pmed1000097

For more information, visit: **www.prisma-statement.org**.

Page 2 of 2
